# Mid-term clinical and radiographic outcomes of porous-coated metaphyseal sleeves used in revision total knee arthroplasty

**DOI:** 10.1186/s43019-021-00103-5

**Published:** 2021-05-04

**Authors:** Ron Gurel, Samuel Morgan, Etay Elbaz, Itay Ashlenazi, Nimrod Snir, Assaf Kadar, Aviram Gold, Yaniv Warschawski

**Affiliations:** 1 Orthopedic Department, affiliated to the Sackler Faculty of Medicine, Tel Aviv Sourasky Medical Center, Ichilov Hospital, Tel Aviv University, 6 Weizman St, 6423906 Tel Aviv, Israel; 2 Tel Aviv University, 6423906 Tel Aviv, Israel

**Keywords:** Revision total knee arthroplasty, Metaphyseal sleeve, Bone loss

## Abstract

**Background:**

The management of bone defects remains one of the major challenges surgeons are faced with in revision total knee arthroplasty (RTKA). Large and uncontained bone defects are traditionally managed with metaphyseal sleeves that facilitate osseointegration and have reported construct stability. While many studies have presented excellent short-term outcomes using metaphyseal sleeves, less is known on their performance in the longer term. The purpose of this study was to present our mid-term results of the metaphyseal sleeves used in patients undergoing RTKA.

**Materials and methods:**

Between January 2007 and January 2015, 30 patients underwent RTKA with the use of a CCKMB prosthesis combined with an osteointegrative sleeve. The main indications for RTKA were instability in 40% of the cases (*n* = 12), aseptic loosening in 30% (*n* = 9), infection in 26.7% (*n* = 8), and “other” in 3.3% (*n* = 1). The minimal follow-up time was 5 years and the mean follow-up time was 82.4 months (SD = 22.6). Clinical outcomes were assessed by Knee Society scores (KSS), range of motion and rate of re-operation.

**Results:**

The mean Knee Society score increased significantly from 72.1 preoperatively to 90.0 postoperatively (*p* < 0.001). The cumulative incidence of re-operation in our study was 13.3% (*n* = 4). Our study reported no cases of aseptic loosening or mobile-bearing spin-out. Knee flexion to 90° and more was impossible in seven cases (23.3%) preoperatively and in one case (3.3%) postoperatively.

**Conclusion:**

Porous-coated metaphyseal sleeves demonstrated excellent rates of survivorship and radiographic ingrowth in the mid-term setting. However, further studies are required to assess their outcomes in the long-term.

## Introduction

The number of revision total knee arthroplasty (RTKA) procedures performed worldwide is increasing, along with the increasing life expectancy [1–3]. The management of bone loss in the femoral and the tibial articular surfaces remains one of the major challenges that surgeons are faced with during RTKA. Metaphyseal bone loss can compromise fixation of components and can lead to component malalignment due to the loss of normal anatomic landmarks [4].

Large and uncontained bone defects are traditionally best managed with structural allografts or metal filling devices such as cones and sleeves [5]. The use of structural allografts has had reported complications of instability, fracture of the graft, as well as reported transmission of infection [6]. Metaphyseal implants therefore represent a promising alternative to address bone loss. While the literature on cones has demonstrated good mid-term outcomes [7–9], their downside involves reported fractures of the host bone, extraction difficulty, and the fact that they may require additional bone grafting [5, 6].

The use of metaphyseal sleeves has gained popularity in recent years due to their purported benefits of construct stability. By facilitating osseointegration, filling bone defects and providing a stable scaffold for knee reconstruction, these sleeves may improve long-term implant survival rate [10]. They are thought to offer advantages over the conventional trabecular cones including optimization of the bone-implant interface and the fact that they add rotational stability, especially in cases of femoral defects with posterior bone loss [11]. While previous studies have demonstrated reliable fixation using sleeves [12–14], these were only evaluated in a short-term setting (< 5-year follow-up).

The literature on the outcomes of metaphyseal sleeves in the mid-term is limited [11, 15, 16] and more evidence is required in order to make definitive clinical decisions. The purpose of our study was to present our mid-term results of metaphyseal sleeves used in patients undergoing RTKA. We hypothesized that that metaphyseal sleeves offer good solution to bone defects encountered during RTKA and have good mid-term survivorship.

## Materials and methods

Institutional Research Ethics Board approval was obtained for this retrospective study (reference no: 0614-19-TLV). A search of our institutional research database was performed to identify patients having undergone revision TKA with a specific CCKMB prosthesis (Press-Fit Condylar Sigma Total Condylar 3 Rotating-Platform; DePuy, Warsaw, IN, USA) between January 2008 and January 2018. Only cases with a minimum of 5 years of follow-up and where an osteointegrative sleeve component was used were included. Data was gathered from the patients’ electronic medical records and included baseline patient characteristics such as gender, age, body mass index (BMI), the use of stem and sleeve components, stem sizes, and the indication for using a constrained implant. The indications for using a constrained implant included instability, aseptic loosening, infection or periprosthetic fracture. Data regarding complications was additionally collected.

**Fig. 1:**
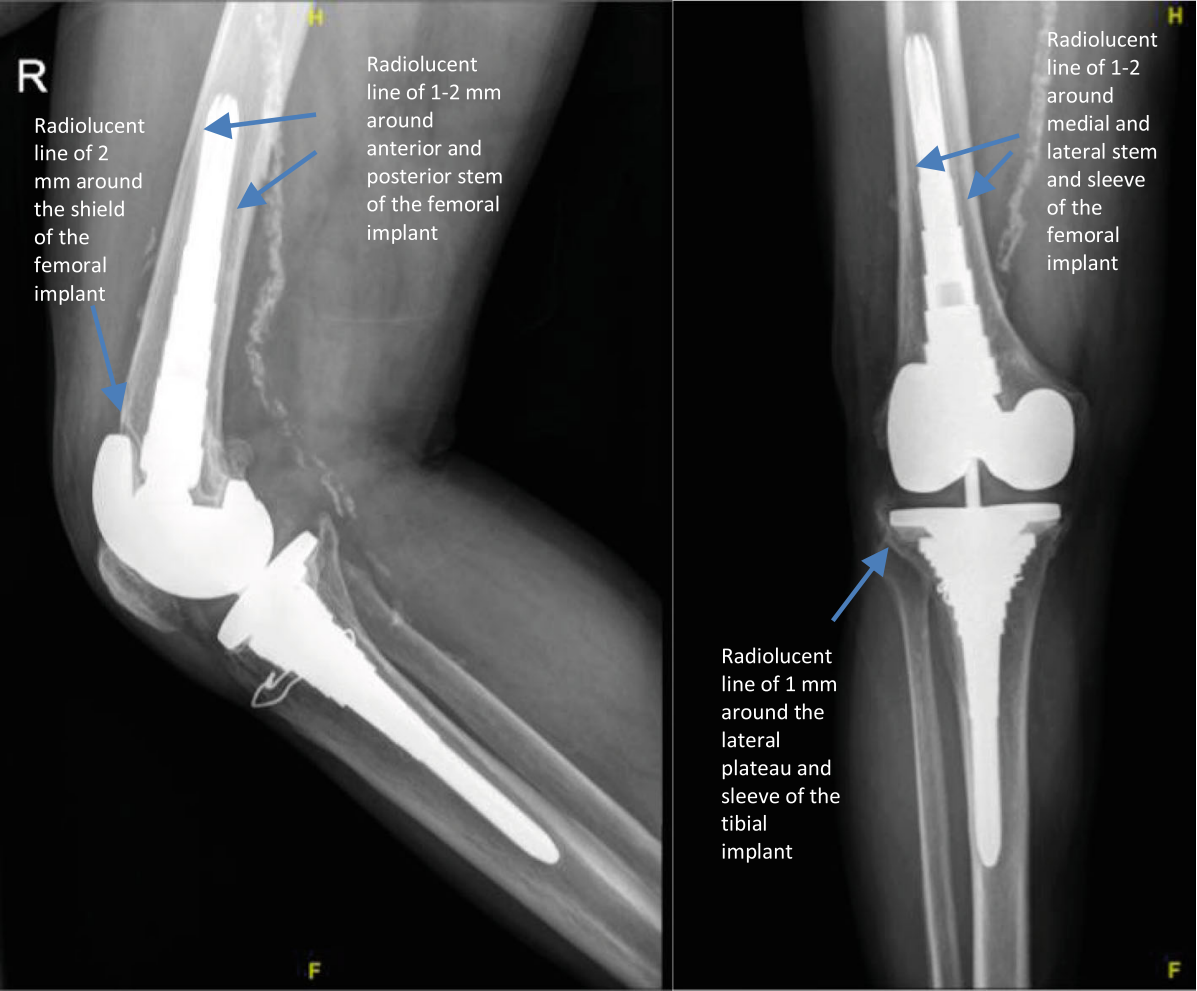
Antero-posterior (AP) and lateral view of the right knee of a 68-year old patient who underwent a second-stage revision total knee arthroplasty. A non-progressive radiolucent line of 1–2 mm around the stem, the anterior part of the femoral sleeve and the tibial plate are seen in radiographs taken 7 years postoperatively. Clinically, the patient had no signs of loosening

All patients were followed at routine postoperative visits at 6 weeks, 3 months, 6 months, 1 year and yearly thereafter. Clinical outcomes were assessed by Knee Society Scores (KSS) [17] and range of motion (ROM). Preoperative and postoperative radiographs, including anterior-posterior, lateral and sky views of the knee, were routinely obtained during follow-up visits in order to evaluate bone defects based on the Anderson Orthopedic Research Institute (AORI) classification. The AORI system classifies femoral and tibial defects separately into types I, II, and III. In type-I defects, the metaphyseal bone is intact, with minor bone defects not compromising component stability. In type-II defects, there is metaphyseal bone damage and cancellous bone loss in one femoral/tibial condyle (type IIA) or both femoral/tibial condyles (type IIB); cement reinforcement, bone grafting or metal augmentation is needed. In type-III defects, the metaphyseal bone is deficient and a structural allograft or a custom-made, hinged or revision prosthesis with an extended intramedullary stem is needed [18]. The radiographs were further evaluated for radiolucent lines graded with the Knee Society rating system [19] and signs of aseptic **l**oosening such as radiolucent lines around the whole implant, implant migration > 2 mm, or cement/implant fracture (Fig 1)**.** Clinical outcomes were assessed by KSS [17], ROM, and documentation of the complications.

Statistical analysis included mean and standard deviation (SD) for continuous variables and percentages and chi square for categorical variables. The *T* test was used to compare mean preoperative to postoperative KSS. Cumulative incidence of re-revision was calculated and presented in Table 5 with descriptions of the causes of re-revisions. Statistical significance was set at a *P* value < 0.05. All analyses were conducted using SPSS software, version 24 (IBM, Armonk, NY, USA).

## Results

Between January 2007 to January 2015, 30 patients underwent RTKA with the use of the CCKMB prosthesis combined with an osteointegrative sleeve. The main indications for RTKA were instability in 40% of the cases (*n* = 12), aseptic loosening in 30% (*n* = 30), infection in 26.7% (*n* = 8), and periprosthetic fracture in 3.3% (*n* = 1) (Table 1). Table 2 describes the characteristics of the study population. Out of 30 cases, 20 (66.7%) were women and 15 (50%) were left knee procedures. The mean BMI was 30.2 kg/m2 (SD = 5.2). The minimal follow-up time was 5 years and the mean follow-up time was 82.5 months (SD = 22.6). Three patients were lost to follow-up. Tables 3 and 4 present the distribution of stems and metaphyseal sleeves use in the femoral and tibial implants of the study population. In 90% (*n* = 27) of the cases a stem was used in both the femoral and the tibial implants and in 70% (*n* = 21) of the cases a metaphyseal sleeve was used in both the femoral and the tibial implants. See Tables 3 and-4 for the full description.

**Table 1:** Indications for revision total knee arthroplasty (TKA)

Indication	*N*	(%)
Instability	12	40.0
Aseptic loosening	9	30.0
Infection	8	26.7
Periprosthetic fracture	1	3.3

**Table 2:** Study population

Age (years)	
Mean (SD)	69.9 (7.0)
Gender, *n* (%)	
Female	20 (66.7%)
Male	10 (33.3%)
Side *n* (%)	
Left	15 (50%)
Right	15 (50%)
BMI (kg/m^2^)	
Mean (SD)	30.2 (5.2)
Follow-up period (months)	
Mean (SD)	82.5 (22.6)
Bone defects, *n* (%)	
Tibia I/IIa/IIb/III	14 (46.7%)/9 (30.3%)/7 (23.3%)/0
Femur I/IIa/IIb/III	28 (93.3%)/2 (6.7%)/0/0

*BMI* Body Mass Index, *SD* standard deviation

**Table 3:** Stem and sleeve use distribution

		*n*	%
Stem	Femoral only	2	6.7
	Tibial only	1	3.3
	Both components	27	90.0
Sleeve	Femoral only	4	13.3
	Tibial only	5	16.7
	Both components	21	70.0

**Table 4:** Stem sizes

Tibia (*n* = 27)	Length *n* (%)	75 mm	22 (81.5%)
		115 mm	5 (18.5%)
	Diameter *n* (%)	10 mm	6 (22.2%)
		12 mm	10 (37.0%)
		14 mm	11 (40.7%)
Femur (*n* = 28)	Length *n* (%)	75 mm	19 (67.9%)
		115 mm	9 (32.1%)
	Diameter *n* (%)	10 mm	1 (3.6%)
		12 mm	12 (42.9%)
		14 mm	13 (46.4%)
		16 mm	2 (7.1%)

### Knee Society Score before and after RTKA

Average preoperative and postoperative KSS (Table 5) of our cohort was 72.1 (SD = 17.3) and 90.0 (SD = 13.9), respectively. Mean difference before and after the operation showed a mean increase of 17.9 points in the score and paired sample *T* test comparing the scores showed this difference was statistically significant (95% CI 8.0–27.8, *P* value < 0.001).

**Table 5:** KSS before and after revision TKA

Preop KSS	Mean (SD)	72.1 (17.3)
Postop KSS	Mean (SD)	90.0 (13.9)
Mean difference: Postop KSS-Preop KSS	Mean (95% CI/*P* value)	17.9 (8.0–27.8/*P* value < 0.001)
Flexion contracture	Preop/postop % (*n*)	23.3% (7)/13.3% (4)
Unable to flex knee to 90°	Preop/postop % (*n*)	23.3% (7)/3.3% (1)

*CI* confidence interval, *KSS* Knee Society Score*, SD* standard deviation, *TKA* total knee arthroplasty

### Ranges of motion before and after RTKA

Preoperatively, seven cases (23.3%) experienced flexion contracture, and postoperatively, the number of cases with flexion contracture decreased to four (13.3%). Knee flexion to 90° and more was impossible in seven cases (23.3%) before RTKA and in one case (3.3%) after RTKA. The mean flexion and extension limits preoperatively were 101.1° (SD = 19.0) and 1.8° (SD = 3.3), respectively. The mean flexion and extension limits postoperatively were 104.6° (SD = 13.6) and 1.5° (SD = 4.3), respectively.

### Radiographic evaluation

Postoperative anteroposterior and lateral radiographs were assessed for radiolucent lines around the femoral and tibial components and the findings are described in Table 6. There were no signs of aseptic loosening in the radiographs of the cohort population.

**Table 6:** Radiolucent lines

	***n***	1 mm	2 mm	3 mm
**Tibial component**				
**Anterior-posterior view**				
Medial plateau	30			
Lateral plateau	30	1		
Medial sleeve	27		1	
Lateral sleeve	27		1	
Medial stem	29		5	1
Lateral stem	29	1		
**Lateral view**				
Anterior plateau	30	1		
Posterior plateau	30			
Anterior sleeve	27		1	
Posterior sleeve	27			
**Femoral component**				
**Anterior-posterior view**				
Medial stem	28	1	2	
Lateral stem	28	1	1	
**Lateral view**				
Shield	30		1	
Anterior sleeve	24		1	
Posterior sleeve	24	2		
Anterior stem	28	2	1	
Posterior stem	28	2	1	

### Re-operation following RTKA with CCKMB implant

Table 7 describes the re-revisions including details regarding the etiology and time of re-revision. Re-revision was required in four patients (13.3%) during the follow-up period. Two of the re-operations were early, as they occurred at 0.3 and 0.7 months after RTKA. One patient was surgically treated for a tibial-tuberosity avulsion fracture at 0.7 months and there was another case of drainage of subcutaneous hematoma at 0.3 months after RTKA. Two re-revisions were late and occurred at 51.9 and 84.2 months after RTKA. Both late re-revisions were the result of patellar clunk.

**Table 7:** Complications

*n*	Time from revision TKA (months)	Complication
1	0.3	Drainage of subcutaneous hematoma
2	0.7	Tibial-tuberosity avulsion fracture
3	51.9	Patellar clunk
4	84.2	Patellar clunk

*TKA* total knee arthroplasty

## Discussion

This study shows satisfying mid-term clinical and radiographic outcomes of porous-coated metaphyseal sleeves used in RTKA. KSS scores significantly increased, ROMs improved, and the re-revision rate was low.

Bone loss is one of the main challenges that surgeons are faced with during RTKA. Large and uncontained bone defects are traditionally managed with trabecular cones and titanium metaphyseal sleeves. Metaphyseal sleeves offer a long-term fixation to the host bone and create a stable platform to receive a femoral or tibial component [20]. As opposed to cones that are bound to the implant through a cement-prosthesis interface, metaphyseal sleeves are attached to the implant through a morse-taper junction, which is thought to remove a source of failure at the cement-implant interface [5, 17]. Furthermore, the added rotational stability in metaphyseal sleeves is thought to offer them an advantage over trabecular cones [21]. Metaphyseal sleeves have reported excellent outcomes in the short-term setting (< 5 years) [12–14].

Bonanzinga et al. [22]performed a systematic review that included 10 studies that used metaphyseal sleeves. From their results, metaphyseal sleeves demonstrated low septic loosening, a low intraoperative fracture rate and, hence, good-to-excellent clinical outcomes. Their review exemplified the excellent osseointegration of sleeves as the aseptic loosening rate for 1413 sleeves in their study was 0.7%. The authors attributed this excellent rate to the high volume-porosity found in metaphyseal sleeves that facilitates bony ingrowth [22]. The mean follow-up time in their study, however, was only 45 months and, as mentioned by the authors, further studies with a longer follow-up are therefore warranted in order to determine the long-term survivorship and the effectiveness of sleeves.

While there is enough evidence to support the use of metaphyseal sleeves in the short term, to date, very few studies have presented their outcomes for a follow-up of more than 5 years [15, 16]. It is, therefore, unclear whether these products are as advantageous in the longer term. The purpose of this study was to present our mid-term results for metaphyseal sleeves used in RTKA. To the best of our knowledge, this study presents the longest mean follow-up time in the literature.

The ROM is one of the most significant factors that influences patient satisfaction after TKA [23]. Knee flexion to 90° and more was impossible in 23.3% of the cases (7 out of 30) before surgery and 3.3% of the cases (1 out of 30) after surgery, demonstrating a markedly improved ROM. Our results moreover demonstrated excellent survivorship of the sleeves as the rate of re-revision during the follow-up period was 13.3% (4 out of 30 knees). Of the cases in our study that required re-revision, none of them were due to aseptic loosening or bearing failure. This aseptic survival rate of 100% at a mean of 82.5 months’ follow-up time was one of the most notable findings in this study and it reflects the inherent stability of the implants.

Our study presents similar findings to the few mid-term studies in the literature that reported good outcomes using metaphyseal sleeves. Fedorka et al. [11] reported a revision rate of 6.8% (5 out of 74 sleeves) in their study over a median follow-up time of 58.8 months. Two point seven percent (2 out of 74 sleeves) required re-operation due to aseptic loosening. In a similar study, Martin-Hernandez et al. [15] reported significant improvements in KSS, Western Ontario and McMaster Universities Osteoarthritis Index (WOMAC), and radiographic assessment over a median follow-up of 71.5 months. Moreover, their study reported no cases of aseptic loosening. In a large cohort study with 104 knees (98 patients, 134 sleeves), Watters et al. [16] reported a metaphyseal sleeve survivorship of 98.5% over a 5.3-year follow-up. Their study additionally reported no cases of aseptic loosening.

In a study by Alexander et al. [24] that presented the short-term results of metaphyseal sleeves, end-of-stem pain was described as a significant complication in metaphyseal sleeves and was reported in seven of their cases (23.3%). As we did not measure this parameter, this presents a limitation in our study.

While there is currently enough evidence to support the use of using metaphyseal sleeves in the short term [22], their impact on patient outcome is less clear in the longer term. The results of our study in conjunction with the outcomes presented in the literature [11, 15, 16] add evidence to support the use of metaphyseal sleeves in the mid-term setting. Further studies assessing the outcomes of these sleeves over a longer duration are necessary in order to assess the long-term survivorship of these implants.

Our study presents with several limitations. Our study population could have been larger and was, therefore, susceptible to a sampling bias. Despite the fact that our study presents outcomes for the longest mean follow-up time described in the literature, no conclusions can be drawn on the performance of sleeves in the long-term setting (> 10 years). Furthermore, as our study lacked a control, it is not possible to definitively support this product over another and future comparative investigations are warranted. Finally, this study’s retrospective nature presents an additional limitation.

## Conclusion

Porous-coated metaphyseal sleeves demonstrated excellent rates of survivorship and radiographic ingrowth in the mid-term setting. Further studies are required to assess the outcomes of metaphyseal sleeves in the long-term.

## Data Availability

The datasets generated during and/or analyzed during the current study are not publicly available due to the intention of the authors to publish additional studies based on these datasets, but they are available from the corresponding author on reasonable request.
